# Massachusetts

**Published:** 1896-04

**Authors:** 


*Massachusetts.* Revised regulations of the Board of Health of the city of Lynn. Adopted March 2, 1896:

Rule 35. On and after May 1, 1896, for all milk brought into or offered for sale in the city of Lynn satisfactory evidence will be required of the producer and dealer by the Board of Health that the milk has been drawn from healthy cows; the condition of health is to be based upon results of tuberculin-test by a veterinarian who is satisfactory to the State Cattle Commission and to the Inspector of Milk for the city of Lynn. After test, each animal to have ear-tag and certificate of health. Also, that the animals used are properly fed and the premises occupied by them are in a good condition of sanitation.

Signed by William L. La Croix, W. B. Little, M.D., Charles Leighton, Board of Health.

